# Non-invasive taxonomic identification of fish species by using scanning electron microscopy: A comparative study of scale microstructures among the *Hypophthalmichthys molitrix* and *Otolithes ruber*

**DOI:** 10.5455/javar.2025.l943

**Published:** 2025-08-18

**Authors:** Arfia Inayat, Zubia Masood, Quratulan Ahmed, Mourad Ben Said, Muhammad Kabir, Muhammad Yousaf, Patricio R. De los Ríos-Escalante, Wajid Ali

**Affiliations:** 1 Department of Zoology, Sardar Bahadur Khan Women’s University, Quetta, Pakistan; 2 Marine Reference Collection and Resources Center (MRCC), University of Karachi, Karachi, Pakistan; 3 Laboratory of Microbiology, National School of Veterinary Medicine of Sidi Thabet, University of Manouba, Manouba, Tunisia; 4 Department of Basic Sciences, Higher Institute of Biotechnology of Sidi Thabet, University of Manouba, Sidi Thabet, Tunisia; 5 Department of Biological Sciences, Thal University Bhakkar (University of Sargodha, Ex-Sub-Campus Bhakkar), Bhakkar, Pakistan; 6 Centre of Animal Sciences & Fisheries, University of Swat, Charbagh, Pakistan; 7 Departamento de Ciencias Biológicas y Químicas, Facultad de Recursos Naturales, Universidad Católica de Temuco, Temuco, Chile; 8 Facultad de Recursos Naturales, Núcleo de Estudios Ambientales, Universidad Católica de Temuco, Temuco, Chile; 9 Facultad de Ciencias, Instituto de Estadística, Pontificia Universidad Católica de Valparaíso, Brasil, Chile; 10 College of Marine Life Sciences, Key Laboratory of Marine Genetics and Breeding, Ministry of Education, Ocean University of China, Qingdao, China

**Keywords:** Non-invasive microstructures, fish scale, taxonomy, *Hypophthalmichthys molitrix*, *Otolithes ruber*

## Abstract

**Objectives:**

Recent studies on teleost fish evolution compare both molecular and morphological data to evaluate the taxonomic relationships. Therefore, the objective of our current study was to assess the potential of using scale microstructures for the taxonomic identification of two fish species, i.e., *Hypophthalmichthys molitrix* and *Otolithes ruber*.

**Materials and Methods:**

Eighty individuals of each carp species were collected from the Quetta fish market between August 2021 and January 2022. Then, microstructures, i.e., scale length and width, focus position, and the number of radii and ctenii of each fish scale obtained from five different fish-body regions, were examined. These measurements were used to compare the variations in scale microstructures between two fish species and may also help evaluate their potential utility in more accurate species identification and systematic classification.

**Results:**

The overall results reveal statistically significant variations (*p* < 0.05) between all examined scale microstructures, including the length or width of the scale, the focus position, and the number of radii or ctenii present on the fish scale, obtained from five body regions of two carp fish species. These results exhibit the potential utility of microstructure studies as a reliable approach for systematic identification. Therefore, our findings support the usage of fish scale microstructures as a valuable and non-invasive tool for taxonomic identification of any fish species. Moreover, this technique may specify the actual impact of these scale features as valuable tools for the conservation and management of endangered or threatened fish species.

**Conclusion:**

Our study provides a foundation for future systematic research and offers a satisfactory approach for non-invasive taxonomic identification.

## Introduction

Fish scales are dermal structures that are found on the fish’s body and are also essential for taxonomic identification due to their species-specific characteristics. Moreover, scales play useful roles in movement and also protect against the impact of various environmental factors, parasites, or predators [1]. The fish scale morphology is a significant tool, which is not only used in taxonomic identification but also helps in analyzing the diets of piscivorous predators and in conducting paleontological studies. The shape and distribution of fish scales across different body regions can help in distinguishing fish species and their populations [2].

Variations in the shape of fish scales have been widely utilized for identifying and differentiating fish populations by various researchers, such as Hina et al. [3] and Feeney et al. [4]. Since fish scales exhibit significant differences in size, shape, structure, and arrangement within the same or different body regions, a wide variety of scale shapes, including elliptical, rectangular, triangular, pentagonal, square, oblong, and circular, have been observed in many bony fishes [2, 3].

Not only fish scales but also other microstructures, including, e.g., scale shape, size, number of ctenii, shape and type of ctenii, number of radii, focus position on the scale, number of circuli, presence or absence of chromatophores, and the structure of the lateral line canal opening on the scale, have been used in the taxonomic classification of fish species [5]. Consequently, several recent studies, such as those by Al Jufaili et al. [6] and Teimori et al. [7], have incorporated additional scale features and microstructures into taxonomic keys for fish identification. These microstructures, such as circuli, radii, ctenii, and chromatophores, are commonly used to classify fish into various taxonomic categories, i.e., orders, families, genera, and species, by numerous researchers, including Echreshavi et al. [2], Zhu et al. [8], Kontaş et al. [9], Rawat et al. [10], and Ibáñez et al. [11]. The number and arrangement/space of circuli on a scale may also vary according to the fish growth rate or due to changes in external water temperature [4].

Scanning electron microscopy (SEM) is an advanced technique that has greatly supported the study of fish scale microstructures, which are now widely used in taxonomic and phylogenetic research by various authors, such as Echreshavi et al. [2] and Esmaeili et al. [12]. For example, Ibáñez and O’Higgins [13] reported that fish scale size and shape are species-specific and can therefore be used to determine fish stock relationships. Thus, fish scales represent an excellent, affordable, and readily accessible material that can be utilized for the identification of fish species, regardless of the anatomical region sampled or the availability of other biological materials for systematic studies.

Traditional methods used for the identification of fish species often involve invasive sampling techniques, which can pose a threat to endangered and vulnerable populations. Recently, non-invasive approaches, such as analyzing fish scales without harming or killing fish, have gained considerable attention. However, there remains a notable shortage of published literature focusing on the use of fish scale morphology and its microstructures as a non-invasive approach for taxonomic identification or phylogenetic analysis, particularly in species such as silver carp (*Hypophthalmichthys molitrix*) and tiger tooth croaker (*Otolithes ruber*). To address this knowledge gap, the present study was conducted to investigate variations in scale microstructures, including, i.e., scale length and width, focus position, and the number of radii and ctenii among these two species, and to evaluate their potential as valuable systematic tools for species classification.

This study is novel in its approach to leveraging scale microstructures for taxonomic and phylogenetic purposes. While traditional morphological and genetic methods are commonly used for species identification [3, 5, 7], they often require invasive techniques that can harm the specimens. Therefore, our current research offers a non-invasive alternative technique that could be particularly valuable for studying endangered or ecologically significant species. Furthermore, the comparative analysis of microstructures across different body regions has not been previously explored for these two species, making this study a pioneering effort in this domain.

## Materials and Methods

### Ethical approval

In the present study, we have used only dead fish samples; however, all study protocols and methods used in the current study had received approval (with approval number F18-MPhil-Zol-1577) from the Departmental Research Ethics Committee of the Zoology Department of Sardar Bahadur Khan Women’s University, Balochistan.

### Fish sample collection

In this study, a total of 160 fish samples, 80 samples each of *O*.* ruber* and *H*. *molitrix*, were collected from a local fish market located near the joint road of Quetta city from August 2021 to January 2022. The size (TL) of each fish sample was measured in centimeters by scaling (Table 1).

### Fish scale slide preparation

**Figure 1.**
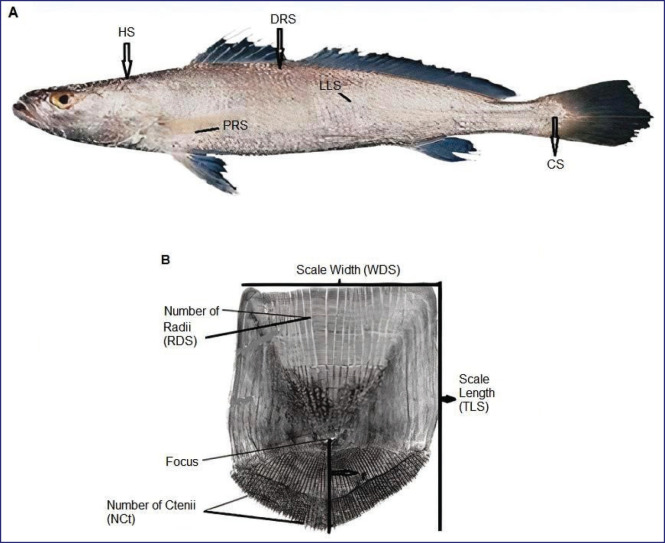
Fish scale analysis: (A) Distribution of scales collected from five different body regions of fish and (B) Microstructures of fish scales.

To observe the microstructures of fish scales, microscopic slides were prepared using the procedures described by Masood et al. [14, 15]. Fish scales were removed from five different body regions of each fish sample, as shown in Figure 1A: HS = scales from the head region, LLS = scales from the lateral line region, CS = scales from the caudal region, DRS = scales below the dorsal fin region, and PFS = scales from the pectoral fin region.

Five to six scales were collected from each body region of the fish using forceps and were soaked in hot water containing 10 drops of 10% NaOH solution. A soft painting brush was used to remove the mucus and dust particles from the surface of each scale. Then, the scales were transferred to different grades of ethyl alcohol solutions (30%, 50%, 70%, and 90%) for dehydration. The scales were then dried using filter paper. A glycerin drop was added to each scale placed on a microscopic slide to prevent it from drying. Another slide was placed on top of the first slide, pressed gently to prevent them from curling after drying, and kept for an hour. A paper tape was wrapped on both ends of each slide for tagging with a code for further study of scale structure under a Leica A60 stereomicroscope and SEM Model JSM-7610F.

Each scale parameter was measured in millimeters (mm), and their abbreviations were as follows: TLS, total length of scale; WDS, width of scale; r, focus position or scale radius; nCt, number of ctenii on scale; and RDS, number of radii on scale (Fig. 1B).

### Microscopic analysis of fish scales using SEM

For the analysis of fish scales using SEM, five clean and dehydrated scales were prepared and stored between two microscopic glass slides for 6 h to dry and prevent curling. The scales were then mounted on SEM stubs using double-sided self-adhesive carbon stickers and were coated with a 100 Å thick layer of gold using a Polaron E 5100. Despite using an SEM (LEO 1430VP) at 15 kV, five images of each scale were captured. The microstructures of each scale were observed at an excessive magnification of over × 2,000 using the technique developed by Masood et al. [14].

**Table 1.** Size of *H. molitrix *and *O. ruber*.

Fish species	L (cm)		W (gm)		L range		W range	
	Mean ± S.D	S. E	Mean ± S.D	S. E	Min.	Max.	Min.	Max.
*H. molitrix*	36.70 ± 2.39	0.379	771.5 ± 48.2	23.4	32.0	40.0	546.0	1062.0
*O. ruber*	28.60 ± 1.446	0.229	246.9 ± 20.1	3.17	26.0	31.0	220.0	292.0

**Note**: Number of fish samples for each species (*N*) = 80; S.E = Standard Error of mean; S.D = standard deviation of mean.

### Statistical analysis of data

All data were analyzed using MS Excel and the statistical software Minitab version 17.1.

**Table 2.** Abbreviations used for five scale parameters and five different fish body parts.

Scale characters	Abbreviation’s
Total length of fish body	L
Length of fish scale	TLS
Width of fish scale	WDS
Number of ctenii on scale	nCt
Total number of radii count on scale	RDS
Position of focus	r
**Five fish body regions for collection of scales**	
Head region scales	HS
Caudal region scales	CS
Pectoral-fin scale	PFS
Dorsal body region scale	DRS
Lateral line region scales	LLS

## Results

### Scanning electron microscopy (SEM) analysis of fish scale microstructures

The descriptive statistical analysis of each scale parameter, including the length of scale (TLS), WDS, focus position (r), number of radii (RDS), and number of ctenii (nCt), was recorded in Table 2. During the SEM study of scales in both species, it was observed that *O*.* ruber* had only ctenoid-type scales, while *H*. *molitrix* had only cycloid-type scales. In this study, only ctenoid scales were found in *O*. *ruber*, which can be attributed to the greater need for protection in marine fishes as compared to freshwater species.

**Figure 2.**
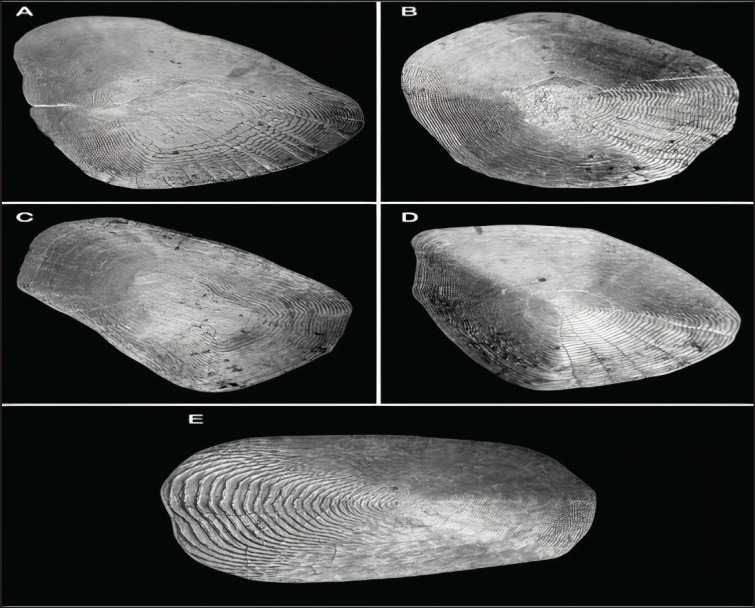
Scales of *H. molitrix*: A = Caudal scale; B = Dorsal region scale; C = Lateral line scale; D = Head scale; E = Pectoral-fin scales.

The fish scale microstructures were also analyzed using SEM in this study. Descriptive statistical analyses were conducted for each fish scale microstructure, including total scale length (TLS), width (WDS), focus position (r), number of radii (RDS), and number of ctenii (nCt), as shown in Table 3. Moreover, the largest scales were observed in *H*. *molitrix*, while the smallest were found in *O. ruber*, which also indicates significant variations in scale size between these two fish species (Figs. 2, 5).

**Figure 3.**
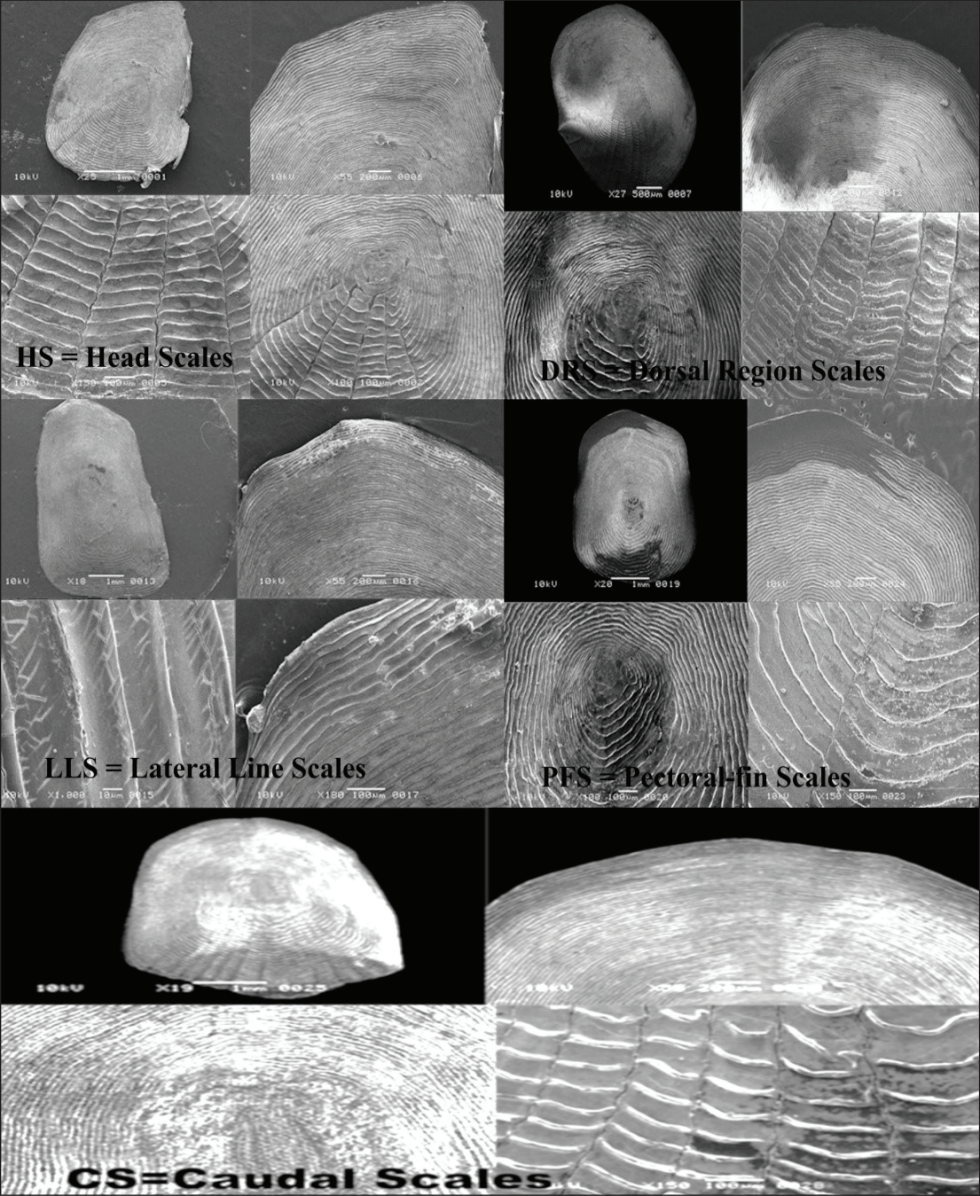
Scanning electron microscopy (SEM) photographs of HS = head scales; DRS = dorsal region scales; LLS = lateral line scales; PFS = pectoral-fin scales; CS = caudal scales of *H. molitrix*.

**Figure 4.**
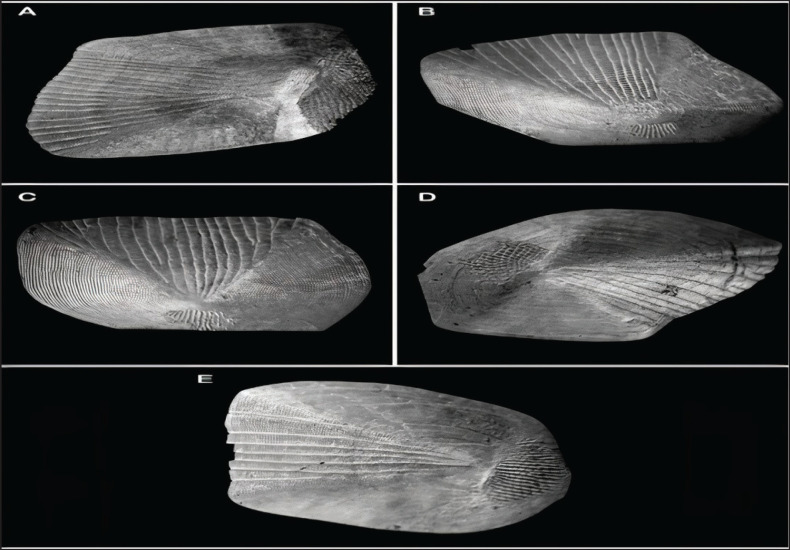
Scales of *O. ruber*: A = Caudal scale; B = Dorsal region scale; C = Lateral line scale; D = Head scale; E = Pectoral-fin scales.

**Figure 5.**
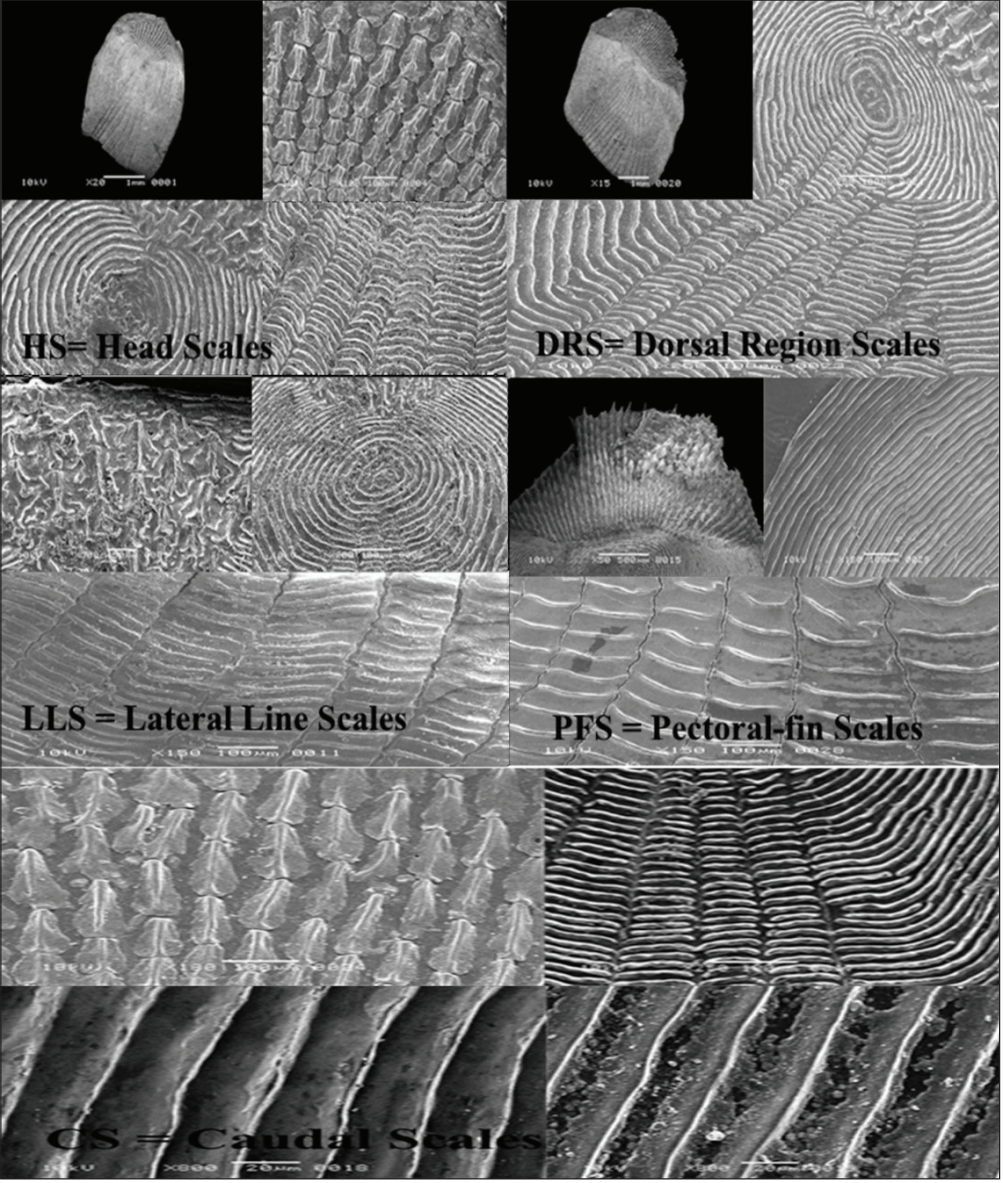
Scanning electron microscopy (SEM) photographs of HS = head scales; DRS = dorsal region scales; LLS = lateral line scales; PFS = pectoral-fin scales; CS = caudal scales of *O. ruber*.

Furthermore, our present study revealed that the fish scale width in *O*. *ruber* was significantly greater than that in *H*. *molitrix*. A great variation in scale size was also observed among the five selected body regions of the fish. For example, the head scales were much smaller in size than those found in the other four body regions of *H*. *molitrix*. Additionally, a notable variation was observed in the number of radii across the five different body regions. The head scales contained only a few radii, whereas the scales from the caudal, pectoral fin, dorsal, and lateral line regions had a large number of radii (Figs. 2, 3). Although the lateral line scales were much larger than the scales in other body regions, their radii were not clear and were rough due to friction during swimming. Conversely, the caudal region scales, although smaller in size, had large numbers of radii that were clearly visible (Fig. 3). The head and caudal region scales of *O*. *ruber* were small, with both ctenii and radii being clearer and more numerous than those obtained from the dorsal, pectoral, and lateral line regions Figs. 4, 5). Therefore, the study showed remarkable variances between the scales of these two species in various scale microstructures, such as scale length and width, focus position, and the number of radii. In *H*. *molitrix*, the scales were small and round, and the focus was more toward the posterior-central position, while in *O*. *ruber*, the scales were large and oblong, and the focus was found in the center of the scale (Figs. 2, 5).

The number of radii was dependent on the speed of fish movement during swimming, and thus the number of radii was larger in the scales of *O*. *ruber* and ranged from 19 to 40 (Fig. 5), while smaller in the scales of *H*. *molitrix*, ranging from 0 to 20, indicating that *O*. *ruber* was a faster swimmer than *H*. *molitrix *(Fig. 3). The ctenii were present only in *O*. *ruber *and ranged from 30 to 40 in number. The position of scales in different body regions of fish is an indication of the condition of the external environment. Therefore, the scales obtained from the lateral line region and the dorsal and pectoral fin regions were rougher in structure than the head and caudal scales. Moreover, both head and caudal region scales were smaller in size than the dorsal, pectoral, and lateral line region scales (Figs. 4, 5).

### Key contribution

This study demonstrates the potential of analyzing fish scales for non-invasive taxonomic identification of fish species. The results highlight the importance of non-invasive techniques for the conservation of endangered or threatened fish species and provide a basis for future research in this field.

## Discussion

As the taxonomic identification for any fish species is quite necessary for the management and conservation of its biodiversity. As a result, since the 1900s, scales of fish have been widely used for genus or species identification, and their several microstructures have also been used as discriminating features or taxonomic keys for them. Therefore, Ibáñez et al. [5] applied the scale characters for the identification of genus, species, or local populations of the family Mugilidae and found that the use of fish scale shape displays guarantees stock discrimination and may lead to a significant or practical fisheries management tool. Scale shapes offer identification tools that could be considered as a rapid, reliable, and cheap material for on-the-spot identification. While the use of otoliths or genital materials for this purpose is more labor-intensive and expensive, it requires equipment [16].

With the advancement in both optical microscopy and SEM in the 20th century, scale microstructures have been assumed to be significant features that could be used in fish taxonomy or classification. Therefore, scanning electron microscopy (SEM) studies of fish scales and their microstructures appear to have a prominent value in the identification of fish species by several workers, including Teimori et al. [7] and Jawad and Al-Jufaili [17].

Furthermore, new details of microstructural features of fish scales have provided remarkable value in the correction of identification and classification of fish [11, 12]. Esmaeili et al. [12] used scale surface microstructures to classify different fish groups at the species level. Their findings revealed that fish scale shape may also vary based on fish habitat, age, and size. Similarly, Viertler et al. [1] analyzed variations in fish scale morphometric characteristics, such as scale size and shape and ctenii coverage, among 240 cichlid species of the family Cichlidae from Africa’s Lake Tanganyika. These variations were attributed to variances in their habitat use, feeding ecology, and fish body shape. Their study highlighted that scale size is strongly correlated with phylogeny, whereas scale shape and ctenii coverage primarily indicate taxonomic group and ecotype, reflecting an adaptive constituent. In addition, they also proposed that scale measurements could assist in connecting individual fish scales with specific taxonomic groups, phylogenetic histories, or ecotypes, including fossils. Ibanez et al. [11] examined site-specific variations in the morphometric characteristics of fish scales collected from five *Cyprinidae* species in New Zealand and Turkey. Echreshavi et al. [2] used optical light microscopy and SEM to study scale features of *Garra sharq* and found that the types, sizes and shapes of scales, lateral surface, focus position, circuli appearance, radii type, lepidonts, and posterior and anterior margin shapes can effectively contribute a key role in the identification and classification of three size classes of an endemic cyprinid fish species, i.e., *G. sharq,* from the Arabian Peninsula. Their study demonstrated that these fish scale characteristics could serve as an alternative tool for the identification, classification, and phylogenetic analysis of different freshwater fish species and genera. Similarly, in our present study, a comparative analysis of various scale microstructures of *O. ruber* and *H. molitrix* was presented to examine valuable scale microstructures that could be later used for observing the differences in these scale characters between the interspecific and within the same species among the five different body regions of the two fish species. Like earlier workers, both *H*. *molitrix* and *O*.* ruber* of this study can also be classified or identified by using some microstructures that might be seen or not in any other fish species. A detailed morphological study of scales in these two species has shown a certain degree of variation in scale shapes and types, focus position, the number of ctenii, and the number of radii found on their scale. The arrangement of the circuli depends on fish scale shapes, and the inter-circulus space is maximum in the lateral and least in the anterior field on the scale. The formation of radii is also correlated with the surface area of both the anterior and lateral fields of fish scales. Moreover, if the fish scale is more flexible or the nutritional condition of the fish is much better, then the number of radii would be increased accordingly [17]. The position focus in scales of *H*.* molitrix* (silver carp) of the present study was very clear and sharp in the scales below the dorsal fin and lay more toward the anterior part of the fish scale, like in the scales of some other carp fishes, i.e., *Tor putitora* and *Catla,* as previously reported by Jawad and Al-Jufail [17]. Thus, our study showed some valuable scale characters, which provide evidence that could be supportive in their systematic study.

**Table 3.** Descriptive statistical analysis of each scale character of two fish species (*H. molitrix *and *O. ruber*)*.*

Variable	*H. molitrix* (*N* = 80)				*Otolithes ruber* (*N* = 80)			
	Mean ± S. D	S.E	Min.	Max.	Mean ± S. D	S. E	Min.	Max.
	Head region scales (HS)							
L	367.0 ± 23.9	3.79	320.0	400.0	286.0 ± 14.4	2.29	260.0	310.0
TLS	2.6 ± 0.5	0.08	2.0	4.0	3.7 ± 1.0	0.16	2.0	5.0
WDS	2.2 ± 0.4	0.06	1.5	3.0	2.9 ± 0.6	0.09	2.0	4.0
r	1.3 ± 0.2	0.04	1.0	2.0	1.8 ± 0.5	0.08	1.0	2.5
nCt	0.0 ± 0.0	0.00	0.0	0.0	5.7 ± 7.8	1.24	0.0	20.0
RDS	3.4 ± 1.1	0.18	1.0	6.0	6.4 ± 4.1	0.65	0.0	13.0
	Caudal region scales (CS)							
L	367.0 ± 23.9	3.79	320.0	400.0	286.0 ± 14.4	2.29	260.0	310.0
TLS	4.4 ± 0.5	0.08	3.0	3.0	4.1 ± 0.6	0.10	3.0	5.0
WDS	3.6 ± 0.5	0.08	3.0	5.3	4.1 ± 0.6	0.10	3.0	5.0
r	2.2 ± 0.2	0.04	1.5	2.5	2.0 ± 0.3	0.058	1.2	2.5
nCt	0.0 ± 0.0	0.00	0.0	0.0	34.8 ± 10.3	1.64	0.0	56.0
RDS	7.8 ± 3.6	0.57	2.0	16.0	21.4 ± 3.5	0.56	14.0	29.0
	Pectoral-fin scales (PFS)							
L	367.0 ± 23.9	3.7	320.0	400.0	286.0 ± 14.4	2.2	260.0	310.0
TLS	4.7 ± 0.5	0.1	4.0	6.0	4.6 ± 0.6	0.1	3.5	6.0
WDS	3.4 ± 0.5	0.1	2.5	4.5	4.3 ± 0.8	0.1	3.0	6.0
r	2.4 ± 0.3	0.0	2.0	4.0	2.2 ± 0.3	0.0	1.5	3.0
nCt	0.0 ± 0.0	0.0	0.0	0.0	7.7 ± 8.4	1.3	0.0	25.0
RDS	1.4 ± 2.0	0.3	0.0	9.0	16.1 ± 4.1	0.6	10.0	28.0
	Dorsal body region scales (DRS)							
L	367.0 ± 23.9	3.7	320.0	400.0	286.0 ± 14.4	2.2	260.0	310.0
TLS	2.9 ± 0.5	0.0	2.0	4.0	3.7 ± 0.7	0.1	2.0	5.0
WDS	2.9 ± 0.6	0.1	2.0	5.0	3.3 ± 0.9	0.1	2.0	5.0
r	1.4 ± 0.2	0.04	1.0	2.0	1.8 ± 0.4	0.0	1.0	2.5
nCt	0.0 ± 0.0	0.0	0.0	0.0	8.9 ± 10.3	1.6	0.0	33.0
RDS	5.7 ± 2.0	0.3	1.0	10.0	16.4 ± 5.5	0.8	6.0	26.0
	Lateral line region scales (LLS)							
L	367.0 ± 23.9	3.7	320.0	400.0	286.0 ± 14.4	2.2	260.0	310.0
TLS	4.7 ± 0.4	0.07	4.0	5.5	4.0 ± 0.9	0.1	3.0	6.0
WDS	3.8 ± 0.4	0.07	3.0	5.0	4.1 ± 0.7	0.1	3.0	6.0
r	2.3 ± 0.2	0.03	2.0	2.7	1.9 ± 0.4	0.0	1.2	3.0
nCt	0.0 ± 0.0	0.0	0.0	0.0	9.0 ± 8.7	1.3	0.0	25.0
RDS	2.3 ± 2.5	0.4	0.0	11.0	18.2 ± 5.1	0.8	6.0	28.0

**Note**: SD = standard deviation of mean, SE = standard error of mean.

Al-Awadhi et al. [18] analyzed the variations in the scale microstructures, such as the inner and outer lateral circuli, inter-radial circuli, and the shape of their denticles, inter-radial and inter-circular grooves, and shapes of ctenii on scales among three fish species (*Epinephelus latifasciatus*, *Epinephelus bleekeri*, and *Epinephelus coioides*) found in the Arabian Gulf of Kuwait. Teimori [19] conducted a study that examined the great diversity in scale shapes, focus position, primary radii, and a few spine shapes in the posterior field of scales within the same species or populations of morphologically related *Aphanius* species, namely, *Aphanius stoliczkanus* and *Aphanius hormuzensis*, gathered from the southern part of Iran. The study indicated that these variations were influenced by environmental factors and genetic variations. Mekkawy et al. [20] studied the variation in some scale characteristics, such as the number of radii and the shapes of ctenii, among different body regions in three *Lutjanus* species (*L. bohar*, *L.* *ehrenbergii*, and *L. monostigma*) collected from the Red Sea of Egypt. They observed that the variability in scale morphology and its microstructures among these *Lutjanus* species was a significant tool in generic or species discrimination and habitat adaptation.

Fish scale mechanical characteristics vary due to factors such as anatomical position, dry and wet conditions, scale anisotropy (which decreases from head to tail), overlapping regions, and temperature fluctuations [21]. The aquatic environment’s climate plays a key role in fish scale morphology and overall body growth. Environmental pollution, including oil exploration and exploitation, can cause significant variation in scale structure across different body regions of a single fish [15]. Bahadur-Dura et al. [22] observed that fish species develop defense mechanisms, including changes in scale shape or thickness, which provide better protection from environmental factors and predators. Wainwright et al. [23] studied scale morphology in 59 species of damselfishes (family Pomacentridae) and found that fish body shape reflects ecological differentiation, which may be linked to scale shape, but not its surface structures. They also discovered a weak evolutionary relationship among various scale morphology traits, but found a strong link between fish scale size and shape. Additionally, they identified an inverse relationship between the number and size of lateral line pores and scale morphology, highlighting the complex evolution of scales. Scale morphology also adapts to different flow environments, with species in open-water habitats having smoother scales. Additionally, the variation in scale shape is considered a significant character for sexual dimorphism in fish populations by Hina et al. [3] and Jawad and AL-Jufail [17]. Mehanna et al. [24] observed the variations in the shapes of lateral line canals found on the scales obtained from *Caranx melampygus* and *Carangoides bajad* as a valuable systematic character for observing the differences between these two fish species found in the Red Sea of Egypt, which was inconsistent with our present study.

Moreover, the weaknesses and limitations of using scale morphology and its microstructures for taxonomic identification and phylogenetic relationships of fishes, and particularly in silver carp (*H. molitrix*) and tiger tooth croaker (*O. ruber*), can be defined as follows: (1) As significant intraspecific variations may exist within individuals of the same species due to environmental factors, growth stages, and diet, potentially confounding the identification process, therefore, use of scale features or microstructures like scale length, width, radii count, focus position, and ctenii distribution might overlap between individuals belong to populations of same species or limiting their diagnostic utility for clear taxonomic distinction. (2) External environmental conditions, such as water temperature, salinity, and habitat, can affect the development of scale microstructures, which may lead to inconsistent results. (3) The observation and measurement of microstructures may involve subjective interpretation, leading to variability in results across different researchers. (4) Although by using this non-invasive technique, scale collection might still cause minor physical damage to the fish, especially in its delicate regions like the head and pectoral fin. (5) Scale morphology may only be reliable for differentiating species within closely related taxa; however, it might be ineffective for distant phylogenetic relationships. Therefore, our current study also addresses these limitations by systematically observing and quantifying variation in scale microstructures across five body regions (head, pectoral fin, dorsal body, caudal, and lateral line) in *H*. *molitrix* and *O*. *ruber*. By focusing on measurable parameters (length, width, focus position, radii, and ctenii), the study seeks to enhance the reliability of scale morphology for systematic purposes and provide insights into its application in species identification.

## Conclusion

Thus, from the comparative study of various scale parameters among two fish species, it was concluded that, like other external body morphological characters of fish, the scale microstructures such as radii, focus, ctenii, scale shape, and size are different in different fish species, and all these microstructures are unique and could also be important for taxonomical applications or some other important applications instead of using some biochemical or biomolecular studies. Moreover, our present work is the first time that describes some new scale microstructures of two different fish species, such as *O. ruber* and *H. molitrix*, which were not investigated before. In addition, these scale characteristics could also be used easily in the taxonomic identification of live, threatened, or endangered species without killing these fish. Therefore, our data could be a valuable resource for taxonomic research and the interpretation of fossil discoveries.

## List of abbreviations

CS, Caudal region scales; DRS, Dorsal body region scale; HS, Head region scales; L, Total length of fish body; LLS, Lateral line region scales; nCt, Number of ctenii on scale; PFS, Pectoral-fin scale; r, Position of focus; RDS, total number of radii count on scale; TLS, Length of fish scale; WDS, Width of fish scale.
